# Use of Intravesical Injections of Platelet-Rich Plasma for the Treatment of Bladder Pain Syndrome: A Comprehensive Literature Review

**DOI:** 10.3390/antibiotics10101194

**Published:** 2021-10-01

**Authors:** Francesco Trama, Ester Illiano, Alessandro Marchesi, Stefano Brancorsini, Felice Crocetto, Savio Domenico Pandolfo, Alessandro Zucchi, Elisabetta Costantini

**Affiliations:** 1 Andrology and Urogynecology Clinic, Santa Maria Terni Hospital, University of Perugia, Umbria, IT Viale Tristano di Joannuccio, 05100 Terni, Italy; ester.illiano@inwind.it (E.I.); marchesi1994@gmail.com (A.M.); elisabetta.costantini@unipg.it (E.C.); 2 Department of Experimental Medicine, University of Perugia, 05100 Terni, Italy; stefano.brancorsini@unipg.it; 3 Department of General and Specialized Surgeries, Renal Transplantation, Nephrology, Intensive Care and Pain Management, University of Federico II, 80100 Naples, Italy; felice.crocetto@unina.it (F.C.); pandolfosavio@gmail.com (S.D.P.); 4 Urology Unit, Department of Translational Research and New Technologies, University of Pisa, 56010 Pisa, Italy; zucchi.urologia@gmail.com

**Keywords:** platelet-rich plasma, interstitial cystitis, bladder pain syndrome, primary bladder pain syndrome, recurrent urinary tract infections

## Abstract

Background: Bladder pain syndrome/interstitial cystitis (BPS/IC) or primary bladder pain syndrome (PBPS) is a complex and poorly understood condition. This comprehensive review aimed to discuss the potential application of platelet-rich plasma (PRP) in the treatment of BPS/IC. The pathophysiology of BPS/IC is characterized by urothelial damage that triggers a chain of events leading to chronic inflammation and other conditions. Frequently, in subjects affected by BPS/IC, recurrent urinary tract infection (rUTI) is associated with difficult therapeutic management. For these reasons, many oral and intravesical treatments (e.g., antibiotic therapy and intravesical anesthetic instillations) have been proposed to alleviate the symptoms of IC/BPS. However, the limitation of these treatments is the short duration of improvement. The purpose of this review is to analyze the efficacy of intravesical PRP injections in subjects with PBS/IC and to try to understand the potential therapeutic effects on the pathophysiology of this disease. Methods: A nonsystematic literature search using Pubmed, EMBASE, Scopus, Web of Science, Medline was performed from January 2000 to August 2021. The following terms were combined to capture relevant publications: “platelet-rich plasma”, “interstitial cystitis”, “PRP”, “bladder pain syndrome”, and “painful bladder syndrome”. Results: After exclusion of non-pertinent studies/articles, we have analyzed 5 studies. In detail, 2 articles concerned preclinical studies in which animal models were used. The authors showed an improvement in the histological pattern with less bleeding in treated subjects, a lower presence of inflammatory cytokines and an increase in the mitotic index of urothelial cells in animals treated with intravesical PRP. In the three prospective clinical trials analyzed, patients with BPS/IC who underwent monthly intravesical PRP injections were found to have a statistically significant improvement in symptoms with modulation of growth factors and inflammatory proteins. Conclusions: New evidence suggests that treatment with intravesical PRP could improve urothelial regeneration and reduces chronic inflammation in BPS/IC, modifying the clinical history of its pathology.

## 1. Introduction

Bladder pain syndrome/interstitial cystitis (BPS/IC) or primary bladder pain syndrome (PBPS) is a chronic inflammatory disorder characterized by pelvic pain for at least 6 months due to increased bladder pain and increased urination frequency, which worsens the quality of life of affected patients [1,2,3,4,5,6,7,8,9,10,11,12,13,14,15,16,17,18,19,20,21,22,23,24,25,26,27,28,29,30,31,32,33,34,35]. The incidence of PBPS is approximately 52–67 per 100,000 cases in the United States of America [2]. Chronic inflammation can cause urothelial dysfunction with edema, the presence of neurogenic inflammatory infiltrates, and ulceration formations [3]. Studies have documented that there is an increase in both tissue and urinary expression of certain neurotrophins, such as the nerve growth factor (NGF), which may indicate a hyperactivation of sensory nerves and increased neuronal plasticity that would lead to the typical sensation of pain present in this pathology [4]. Additionally, there has been a reported increase in inflammatory cytokines, such as tumor necrosis factor- alpha and interleukin (IL)-6 and 8 [5]. These events could lead to a malfunction of urothelial tight junctions with loss of urinary blood barrier integrity and subsequent loss of potassium within the interstitium [6]. Overexpression of some angiogenic growth factors, such as vascular endothelial growth factor (VEGF), has also been demonstrated, which can induce the formation of immature vasculature easily demonstrated during the hydrodistension procedure with the formation of typical glomerulations [7]. Moreover, in patients with BPS/IC, there is a decreased bladder capacity due to the process of organ fibrosis that is generated after chronic inflammation. In these patients, there is an increase in transforming growth factor (TGF)-beta 1 and hyperactivation of matrix metalloproteinases (MMPs) [8].

In addition, in 38% of patients, regardless of clinical phenotype, there was a history of bacteriuria in the previous 2 years [8]. For this reason, many clinicians treated these patients with BPS/IC with antimicrobials, without benefit on the symptomatology, but on the other hand, it posed problems, such as the alteration of the urinary microbiota already altered in BPS/IC and the phenomena of antibiotic resistance, such as difficulty in eradicating a possible concomitant urinary tract infection (UTI). To date, there is no single treatment that is effective in all cases [1]. In addition, many authors suggest proceeding with a single treatment at a time and evaluating the outcome based on whether to replace or add other types of treatment [1]. International guidelines emphasize the concept that procedures should range from the simplest to the most complex therapeutic solutions. In addition, multiple and simultaneous treatments can be used, and if the current treatments become ineffective, they should be discontinued and replaced. Multiple intravesical treatments have been proposed for the treatment of IC/BPS.

Intravesical instillations are based on two fundamental assumptions: restoration of the urothelial barrier or topical anti-inflammatory action [3]. One of the drugs used for topical anti-inflammatory action is Dimethyl Sulfoxide (DMSO) [3]. This substance has proven to be useful due to its anti-inflammatory, analgesic and muscle relaxant properties and is also able to block mast cell degranulation. The main problem of this substance is that it can initially worsen the symptomatological picture and therefore patients are forced to suspend it.

Another intravesical treatment for some years is hyaluronic acid associated or not with chondroitin sulfate. This type of substance acts as a repairer of the urothelial barrier by acting as an exogenous glycosaminoglycan (GAG) and restoring the damaged epithelium, so that the bladder submucosa does not encounter urinary metabolites, including potassium, and trigger an inflammatory reaction that tends to become chronic [4].

One of the main problems with the use of intravesical hyaluronic acid is the cost incurred and the fact that very often the improvement is temporary.

For these reasons, new therapeutic approaches have been attempted that aim at a resolution of the pathology. One of the most recent approaches consists of intravesical injection of platelet-rich plasma (PRP).

PRP is a leukocyte-free autologous plasma mixed with plasma proteins prepared from the patient’s own blood [9]. PRP preparation has been used in many areas of regenerative medicine, such as in orthopedics or maxillofacial surgery, even if its mechanism of action is still unclear [10].

It has been hypothesized that within the PRP, there are some growth factors, such as the platelet-derived growth factor (PDGF), TGF, and epidermal growth factor (EGF), that stimulate mitogenesis, chemotaxis, and cell differentiation [11]. Specifically, it has been hypothesized that PRP injected within the bladder may promote repair of urothelial defects and regeneration of the epithelium that is damaged in patients with IC/BPS [12]. Therefore, the purpose of this review was to discuss the clinical and preclinical evidence published to date regarding the use of PRP in the treatment of IC/BPS.

### Notes on the Pathophysiology of IC/BPS

The pathophysiology of IC/BPS rests on three fundamental elements [13]:

- dysfunction of the epithelial barrier;
- mast cell activation; and
- neurogenic inflammation.

The therapy currently approved for this pathology is essentially based on the aforementioned elements [14]. In fact, the substances that are used can restore the endothelial barrier, and they try to “turn off” the neurogenic inflammation and block the uncontrolled mast cell activation [14]. Following a bladder insult, a vicious circle is generated in which the damage is perpetuated, and in this way, bladder pain syndrome associated with urination disorders develops [15]. Subsequently, bladder fibrosis can be generated, and through the process of neuronal plasticity, chronic neuropathic pain can be established [15].

## 2. Materials and Methods

This comprehensive review was performed because there are still some uncertainties regarding the correct therapy for the treatment of IC/BPS. In addition, there is increasing interest in treatments based on the pathophysiology of the disease, not just symptom management. The characteristics of a usable intravesical substance include penetration of the urothelium, long-lasting effects, and low cost. For these reasons, we focused on relatively recent PRP therapy. The literature search was performed in the Medline (US National Library of Medicine, Bethesda, MD, USA), Scopus (Elsevier, Amsterdam, The Netherlands), and Web of Science Core Collection (Thomson Reuters, Toronto, ON, Canada), Pubmed and EMBASE databases from January 2000 up to August 2021. The following terms were combined to capture relevant publications: “platelet-rich plasma”, “interstitial cystitis”, “PRP”, “bladder pain syndrome”, and “painful bladder syndrome”. The Boolean operator (AND) was incorporated to augment the search. Two authors reviewed the records separately and individually to select relevant publications, and any discrepancies were resolved by a third author.

## 3. Results

**Figure 1:**
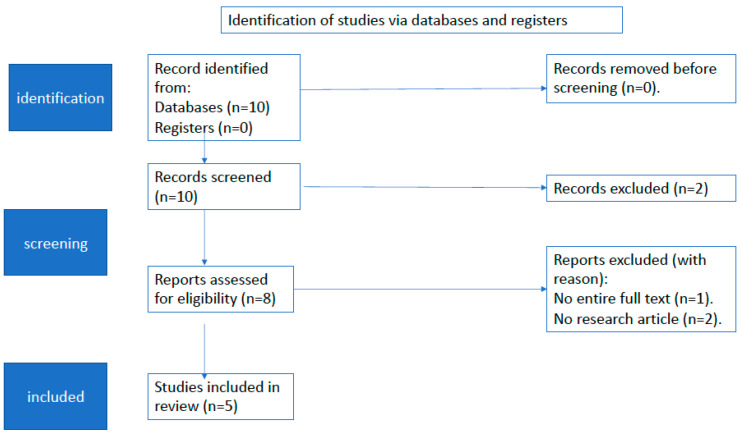
Flow diagram of study selection for this review.

The search strategy yielded 10 results. Screening of the titles and abstracts revealed eight papers that were eligible for inclusion. We initially excluded two articles because they were not written in English. Further assessment of eligibility, based on the full-text papers, led to the exclusion of three papers; one article was excluded because there was no full text and two articles were excluded because they were not in English. Finally, five papers (two preclinical studies and three clinical trials) were included in the final analysis (Figure 1).

### 3.1. Preclinical Studies

Donmez et al. [16]. evaluated the early histological effects of intravesical PRP instillation in rabbit models of interstitial cystitis and hemorrhagic cystitis (Table 1). They divided 36 rabbits into six groups according to the substances injected intravesically: saline solution (S), S + PRP, hydrochloric acid (HCl), HCl + PRP, cyclophosphamide (CyP), and CyP + PRP. At 48 h after induction by HCl, which mimicked the presence of interstitial cystitis, and CyP, which mimicked the presence of hemorrhagic cystitis, the preparation containing PRP was instilled. They found that the mean mitotic index and profiling index were higher in the S + PRP group than in the S group (*p* = 0.004 and *p* = 0.009, respectively). In addition, the mean mitotic index increased (*p* = 0.002) in the HCl + PRP group compared to the HCl group. Furthermore, in the Cyp + PRP group, there was a significant reduction in intravesical bleeding (*p* = 0.006) and an increase in leukocyte infiltration (*p* = 0.038) and the mitotic index (*p* = 0.002). They concluded that intravesical injection of PRP increased the mitotic index and decreased bladder bleeding [16].

In 2020, Chen et al. [17] evaluated the efficacy of intravesical instillation with PRP and hyaluronic acid (HA) in mouse models of IC/BPS, and the possible proliferation of normal human fibroblast cells (HFCs). The authors randomized 30 virgin female rats into five groups according to the intravesical substance injected. In group 1 or the control group, S was instilled; in group 2, Cyp + S; in group 3, Cyp + HA (1 mg/mL); in group 4, CYP + PRP; and in group 5, CYP + PRP + HA. They found that a low dose of PRP increased the proliferation of HFCs (*p* < 0.05). Additionally, the intervals between micturition were greater in the CYP + PRP group than in the CYP + S group (*p* < 0.05). Moreover, the expression of cell junction-associated protein zonula occludens 2 (ZO-2) was increased (*p* < 0.05) and the expression of IL-6 was decreased (*p* < 0.05) in group 4 compared to group 2. Furthermore, they showed that there was no synergistic effect when PRP and HA were administered together (*p* > 0.06), but rather PRP, due to its ability to express ZO-2, would play a predominant role in urothelial repair [17].

In both studies analyzed, PRP was obtained according to the procedure described by Nagae et al. [18]; via the intracardiac waist, blood was drawn and subsequently transferred to a tube containing 3.2% citrate. The sample was subsequently centrifuged at 1500 rpm for 10 min to separate the supernatant from the buffy coat. The remaining plasma was centrifuged at 3000 rpm for 10 min. The supernatant was discarded, and approximately 1 mL of PRP was obtained from the buffy coat [16,17].

### 3.2. Clinical Trial

Jhang et al [19]. investigated the clinical efficacy of intravesical PRP injections in IC/BPS (Table 2). The authors performed four intravesical injections of PRP (volume of 12 mL extracted from 50 mL of the patient’s own blood) at monthly intervals in 15 patients with IC/BPS and analyzed the O’Leary-Sant symptom (OSS) score, as the primary endpoint, at baseline and at 30 days and 4 months later. In addition, they analyzed pain perception using the visual analog scale (VAS), urination frequency, functional bladder capacity, maximum flow rate, post-urination residual (PVR), and global response assessment (GRA), which is a symmetric seven-point scale; the result was considered excellent when patients reported improvement in the score by >2, and the result was considered improved if there was an improvement of >1. They also investigated urinary cytokine levels at baseline and 1 month and 4 months later.

The results of this study at 1 month after the last treatment were as follows. In total, 13 of 15 patients completed the course of intravesical injections with PRP. The VAS and OSS scores decreased significantly (*p* < 0.05) after the first PRP injection, and there was an increase in the GRA score (*p* < 0.05) and an increase in bladder capacity (*p* < 0.05). In addition, there were no changes in post-micturition residual or micturition difficulties (*p* > 0.05), but there was an increase in the expressions of IL-2 (*p* = 0.028) and IL-8 (*p* = 0.019), suggesting an immunomodulatory response following the PRP injections [19].

Subsequently, in a prospective clinical trial with a longer follow-up, Jhang et al. [20]. investigated the efficacy of PRP injections in patients with IC/BPS refractory to conventional therapies. They enrolled 40 patients (37 women and 3 men) and performed four monthly intravesical injections of 10 mL of PRP. In this study, the authors analyzed the GRA score as the primary endpoint at 3 months after the last injection of PRP and the OSS score, VAS score, micturition frequency, nocturia, functional capacity, PVR, and maximum flow rate as secondary endpoints at baseline and at 3 months after the last intravesical injection of PRP. The authors found that 3 months after the fourth treatment, the success rate was 67.5%. Moreover, the OSS and VAS scores, nocturia, and urination frequency decreased significantly (all, *p* < 0.05). PVR did not change significantly, while bladder capacity increased (*p* < 0.05) [20].

Jiang et al. [21]. searched for objective evidence regarding the efficacy of PRP injections in a subsequent study. The previously cited studies emphasized the efficacy of the treatment based on subjective criteria, such as the patient’s perceived symptoms. In Jiang et al.’s study, published in 2020, the urinary levels of 13 functional proteins, including growth factors and cytokines, were investigated at baseline and after the fourth intravesical injection of PRP. The urinary levels of the analyzed proteins after the fourth intravesical injection of PRP had undergone significant changes; that is, the levels of NGF (*p* = 0.043), MMP-13 (*p* = 0.001), and VEGF (*p* < 0.0001) decreased significantly, while the values of PDGF (*p* = 0.004) increased. Furthermore, the authors compared the results obtained with those of a control group whose subjects did not have bladder symptoms. Only the urinary VEGF level was higher in patients with IC/BPS at baseline; however, it was lower after PRP injection in patients with IC/BPS than in the controls (all, *p* < 0.0001) [21].

In the clinical trials, PRP was prepared by taking 50 mL of whole blood and centrifuging it at 190× *g* for 20 min. The supernatant containing platelets was transferred to another tube without touching the buffy coat. The platelet-containing plasma was centrifuged at 2000× *g* for another 20 min. In this manner, at the bottom of the tube, platelet pellets formed; in the upper two thirds. platelet-poor plasma (PPP) formed; and in the lower third, PRP formed. The PPP was removed, and the platelet pellets were added to the plasma to form 10 mL sterile PRP [19,20,21].

An endoscopic procedure consisting of 20 suburothelial injections of PRP was performed in patients with IC/BPS. Each injection corresponded to 0.5 mL of PRP. A 23-gauge needle was inserted 1 mm inside the bladder wall (the lateral and posterior walls were involved), and then hydrodistension was performed up to a bladder capacity of 500 mL to activate the injected substance.

## 4. Discussion

IC/BPS is usually caused by urothelial destruction and increased permeability of the blood-urine barrier, which triggers chronic inflammation; macrophages, mast cells, and eosinophils are drawn in situ [22]. With the persistence of inflammation, there is a loss of integrity of the urothelial mucosa in conjunction with an increase in cell apoptosis and an inability of the urothelium to regenerate effectively [23]. Unfortunately, in therapy, there is a greater attention of researchers on the treatment of symptoms experienced by the patient, and there is still little evidence regarding the treatment of the causes that underlie the pathophysiology of IC/BPS [24]. Since in many patients there is a concomitant diagnosis of bacterial cystitis, they initially sought to demonstrate the efficacy of a broad-spectrum, long-term antibiotic therapy.

Warren et al. showed that a sequential antibiotic therapy taken for several weeks and consisting of rinfampicin, doxycycline, erythromycin, metronidazole, clindamycin, amocxicillin, and ciprofloxacin (each molecule administered for 3 weeks) was associated with a decrease in symptoms compared to placebo (48% vs 24%) at the expense of a significant increase in morbidity due to side effects of the drugs administered [25]. For these reasons, the authors stated that there was no major advantage in antiobicotherapy in patients with BPS/IC [25]. Therefore, the idea of using PRP in IC/BPS has been borrowed from the application of PRP for regenerative medicine in other fields of medicine, such as orthopedics or esthetic medicine [26].

PRP contains bioactive molecules, such as growth factors, cytokines, and molecules, that help tissue repair [25]. In particular, PRP seems to play an important role because it triggers the beginning of a new inflammatory process that would lead to a more rapid resolution of the one generated by the insult that causes IC/BPS and subsequently leads to the repair of damaged tissues [27]. The latter, in contact with PRP, activates the substances contained within it with subsequent release of growth factors and proliferation of cells present in the basement membrane of the urothelium [27].

PRP is composed of substances that were contained within platelets and have already been degranulated and that the body normally uses for the reparative process of wounds [28]. Hence, PRP therapy is based on the proliferation of cells and their migration to the damaged site, subsequent cell differentiation, and angiogenesis [29].

**Figure 2:**
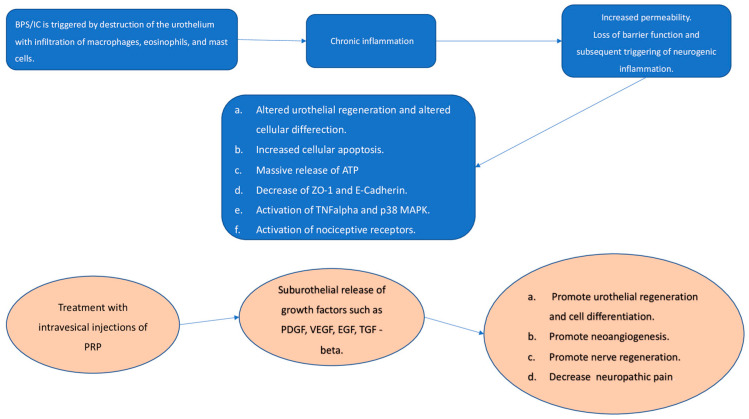
Possible therapeutic effects of intravesical injections of platelet-rich-plasma (PRP). ATP: Adenosine triphosphate; ZO-1: zonulae occludens-1; TNF alpha: tumor necrosis factor; MAPK: mitogen-activated protein kinase; PDGF: platelet-derived growth factor; VEGF: Vascular endothelial growth factor; EGF: Epidermal growth factor; TGF-beta: Transforming growth factor.

Patients with IC/BPS have increased urinary VEGF levels. Peng et al. [21] reported that in subjects with IC/BPS and treated with botulinum toxin A, there was a downregulation of VEGF expression in bladder tissues [28]. These results suggest that VEGF plays an important role in aberrant neo-angiogenesis typical of the disease with the formation of glomerulations and hemorrhage [30]. PRP has been shown to reduce urinary VEGF levels in all treated subjects, leading to an improvement of symptoms [21]. In addition, Jiang et al. demonstrated that there was an increase in PDGF expression in the urine of subjects treated with PRP injections [21]. PDGF has been shown to have pleiotropic effects. This suggests that PDGF is important in repairing urothelial defects, increasing the number of fibroblasts and smooth muscle cells, and promoting tissue oxygenation and subsequent remodeling [26]. These mechanisms, in association with another growth factor contained within the PRP, EGF, promote cell regeneration in the damaged urothelium, and counteract tissue fibrosis created as a result of the disease (Figure 2).

PRP has also been applied to treat soft tissues with marked benefits and repair of difficult-to-treat ulcers [29]. Moreover, PRP appears to be able to modulate neuropathic pain [31]. In rats with spinal cord injury, PRP promoted neuronal regeneration [31]. For this reason, it would seem that once injected into the bladder wall, PRP can help with axonal regeneration and consequently modulate aberrant pain signals coming from the bladder.

In addition, since BPS/IC is associated in many patients with recurrent UTI (rUTI), they tried to justify treatment with intravesical PRP in this cohort of patients as well. Mirzaei et al. analyzed 30 patients with recurrent bacterial cystitis compared to a placebo group and showed that there was a significant decrease in the number of recurrent cystitis episodes and a decrease in the ICIQ-OAB score in the PRP-treated group up to one year after the end of treatment [32]. Yuan-Hong Jiang et al. analyzing 22 patients with rUTI with monthly intravesical PRP injections and 17 controls demonstrated an increase in urothelial cell proliferation and an increase in cytokine (CK) 20 expression in umbrella cells [33].

Intravesical PRP instillation in patients with rUTI as well as in subjects with BPS/IC improves urothelial barrier function by preventing damage caused by uropathogenic bacteria that destroy the epithelium and alter urothelial homeostasis with positive potassium chloride sensitivity test and finally bladder pain [34].

There are several limitations of the studies analyzed: there was no randomized trial, the sample sizes were small, these studies often had short follow-up periods, and the choice of intravesical injections of PRP was arbitrary. As no studies have demonstrated the long-term safety of PRP and the possibility of repeated treatment in the long term, future research should focus on conducting randomized trials with adequate sample sizes and longer follow-up periods.

## 5. Conclusions

Intravesical treatment with PRP, according to the studies published in the literature, could be an innovative treatment, albeit minimally invasive, with efficacy in terms of improving clinical symptoms, and preclinical and clinical evidence suggests that PRP can change the natural history of the disease, leading to its clinical and pathophysiological improvement. However, further studies are needed to demonstrate its true clinical efficacy. In addition, studies indicate that PRP can also be used to treat BPS/IC-like urologic conditions such as rUTI.

## Figures and Tables

**Table 1:** Main features of the preclinical studies analyzed.

Study	Donmez et al., 2016 [16]	Chen et al., 2020 [17]
Purpose	To investigate the early histological effects of intravesical instillations of PRP	To investigate whether the intravesical instillation of PRP could be ameliorate urothelial injury
Animal model used	36 rabbits	30 virgin female rats
Design	Experimental	Experimental
Investigated variables	Histopathology of the bladder sections, mitotic index	Histopathology of the bladder sections, ZO-2 expression, IL-6 expression, voiding intervals value
Significant results	In the PRP-treated groups, there was an increase in the mitotic index, reduction in hemorrhage, and an increase in leukocyte infiltration	In the PRP-treated groups, there was an increase in HFCs, an increase in the interval between micturitions, an increase in ZO-2 expression, and a decrease in IL-6 expression

PRP: platelet-rich plasma; ZO-2: zonula occludens 2; IL-6: interleukin 6; HFCs: human skin fibroblast cells.

**Table 2:** Main features of the clinical trials analyzed.

Study	Jhang et al., 2017 [19]	Jhang et al., 2018 [20]	Jiang et al., 2020 [21]
Purpose	To investigate the clinical efficacy of intravesical injections of PRP	To investigate the clinical efficacy of intravesical injections of PRP	To investigate the changes in urinary markers after PRP treatment
Design	Prospective clinical trial	Prospective clinical trial	Prospective clinical trial
Subjects	*N* = 15 women, but 13 completed the 4 injections and follow-up visits	*N* = 40 (37 women and 3 men)	*N* = 40 (37 women and 3 men)
Protocols	4 PRP endovesical injections monthly	4 PRP endovesical injections monthly	4 PRP endovesical injections monthly
Primary outcome	Change in the OSS index from baseline to 1 month after the 4th PRP injection	GRA score at 3 months after the 4th PRP injection	Urinary levels of cytokines, functional proteins, and growth factors
Secondary endpoints	VAS score, daily urination frequency, nocturia, FBC, maximum flow rate, voided volume, PVR, GRA score, IL-2 expression, and IL-8 expression	OSS index, VAS score, daily urination frequency, nocturia FBC, maximum flow rate, voided volume, PVR	OSS index, VAS score, daily urination frequency, nocturia FBC, maximum flow rate, voided volume, PVR
Significant results	OSS index and VAS score decreased, FBC and GRA score increased after the 1st PRP injection until 1 month after the 4th PRP injection, no change in PVR	GRA score improved after the 1st PRP injection until 3 months after the 4th PRP injection, OSS index and VAS score decreased, FBC decreased and urination frequency and nocturia decreased, no change in PVR	GRA score improved; VAS score, urination frequency, and nocturia decreased; urinary levels of NGF and VEGF decreased; urinary level of PDGF increased

PRP: platelet-rich plasma; *N*: number; OSS: O’Leary-Sant Symptom index; GRA: global response assessment; VAS: visual analog scale; FBC: functional bladder capacity; PVR: post-void residual; NGF: nerve growth factor; VEGF: vascular endothelial growth factor; PDGF: platelet-derived growth factor.
